# Supplementary material for: Monitoring Emergence of the SARS-CoV-2 B.1.1.7 Variant through the Spanish National SARS-CoV-2 Wastewater Surveillance System (VATar COVID-19)
Source: Environ Sci Technol. 2021 Aug 16;55(17):11756–66. doi: 10.1021/acs.est.1c03589 (PMC8404293; doi:10.1021/acs.est.1c03589)
Supplement: Supplementary file 1 — es1c03589_si_001.pdf [file es1c03589_si_001.pdf]

SUPPORTING INFORMATION

Monitoring emergence of the SARS-CoV-2 B.1.1.7 Variant through the Spanish National SARS-CoV-2 Wastewater Surveillance System (VATar COVID-19)

Albert Carcereny[1,2†], Adán Martínez-Velázquez[1,2†], Albert Bosch[1,2], Ana Allende[3], Pilar Truchado[3], Jenifer Cascales[3], Jesús L Romalde[4], Marta Lois[4], David Polo[4], Gloria Sánchez[5], Alba Pérez-Cataluña[5], Azahara Díaz-Reolid[5], Andrés Antón[6], Josep Gregori[7,8], Damir Garcia-Cehic[7,8], Josep Quer[7,8], Margarita Palau[9], Cristina González Ruano[10], Rosa M Pintó[1,2#*] and Susana Guix[1,2#*]

[1] Enteric Virus laboratory, Department of Genetics, Microbiology and Statistics, Section of Microbiology, Virology and Biotechnology, School of Biology, University of Barcelona, Barcelona, 08028, Spain.

[2] Research Institute of Nutrition and Food Safety (INSA), University of Barcelona, Santa Coloma de Gramenet, 08921, Spain.

[3] Research Group on Microbiology and Quality of Fruit and Vegetables, CEBAS-CSIC, Murcia, 30100, Spain.

[4] Department of Microbiology and Parasitology, CIBUS-Faculty of Biology & Institute CRETUS, Universidade de Santiago de Compostela, Santiago de Compostela, 15782, Spain.

[5] Department of Preservation and Food Safety Technologies, Institute of Agrochemistry and Food Technology, IATA-CSIC, Paterna, 46980, Spain.

[6] Microbiology Department, Vall d'Hebron Institut de Recerca (VHIR), Vall d'Hebron Hospital Universitari, Vall d'Hebron Barcelona Hospital Campus, Barcelona 08035, Spain.

[7] Liver Unit, Liver Diseases - Viral Hepatitis, Vall d'Hebron Institut de Recerca (VHIR), Vall d'Hebron Barcelona Hospital Campus, Barcelona, 08035, Spain.

[8] Centro de Investigación Biomédica en Red de Enfermedades Hepáticas y Digestivas (CIBERehd), Instituto de Salud Carlos III, Madrid, 28029, Spain.

[9] General Directorate of Public Health, Ministry of Health, Madrid, 28014, Spain

[10] Subdirección General de Protección de las Aguas y Gestión de Riesgos, Ministerio para la Transición Ecológica y el Reto Demográfico, Madrid, 28071, Spain

† Equally contributed

# Address correspondence to Susana Guix (susanaguix@ub.edu) and Rosa Pintó (rpinto@ub.edu)

* Co-corresponding authors

This document contains 4 pages and 2 Tables.

Table S1. Parameters defining standard curves and limit of detection (LOD) and limit of quantification (LOQ) (genome copies/reaction, GC/rxn) for N1 target and S duplex RT-qPCR assay, of all 4 participating laboratories.

Lab	Target	Slope	Intercept	Efficiency	R^2	LOD (GC/rxn)	LOQ (GC/rxn)
A	N1	-3.696	42.45	86.45	0.981	11.23	28.45
	S_Probe6970in	-3.702	40.22	86.24	0.977	6.07	27.61
	S_Probe6970del	-3.615	40.31	89.07	0.986	6.39	24.76
B	N1	-3.473	39.46	94.10	0.946	10.33	36.13
	S_Probe6970in	-3.734	41.47	85.28	0.967	8.00	21.59
	S_Probe6970del	-3.430	41.29	95.69	0.989	5.60	7.92
C	N1	-3.667	39.99	87.37	0.967	8.14	12.69
	S_Probe6970in	-3.867	42.47	81.38	0.996	29.58	30.60
	S_Probe6970del	-3.612	39.43	89.18	0.989	2.63	14.76
D	N1	-3.169	37.38	105.85	0.977	5.74	19.90
	S_Probe6970in	-3.054	37.97	112.52	0.983	3.91	7.40
	S_Probe6970del	-3.409	38.62	96.40	0.996	5.60	15.31

Table S2. Time (weeks) required for B.1.1.7 variant to reach a 90-100% prevalence for at least 2 consecutive weeks.

WWTP	City	Weeks
4	Granada	5
10	Sevilla	9
13	Bilbao	9
20	Santander	7
21	Segovia	10
24	Albacete	10
25	Guadalajara	10
27	Igualada	8
36	Cáceres	5
